# Whole-Body Distribution of Donepezil as an Acetylcholinesterase Inhibitor after Oral Administration in Normal Human Subjects: A ^11^C-donepezil PET Study

**DOI:** 10.22038/aojnmb.2016.7513

**Published:** 2017

**Authors:** Ikuko Mochida, Eku Shimosegawa, Yasukazu Kanai, Sadahiro Naka, Keiko Matsunaga, Kayako Isohashi, Genki Horitsugi, Tadashi Watabe, Hiroki Kato, Jun Hatazawa

**Affiliations:** 1 Department of Nuclear Medicine and Tracer Kinetics, Osaka University Graduate School of Medicine, Osaka, Japan; 2 Osaka University Graduate School of Medicine, Immunology Frontier Research Center, Osaka, Japan; 3 Department of Molecular Imaging in Medicine, Osaka University Graduate School of Medicine, Osaka, Japan; 4 Osaka University Hospital, Osaka, Japan

**Keywords:** ^11^C-DNP PET, Donepezil, Oral dosing

## Abstract

**Objective(s):**

It is difficult to investigate the whole-body distribution of an orally administered drug by means of positron emission tomography (PET), owing to the short physical half-life of radionuclides, especially when ^11^C-labeled compounds are tested. Therefore, we aimed to examine the whole-body distribution of donepezil (DNP) as an acetylcholinesterase inhibitor by means of ^11^C-DNP PET imaging, combined with the oral administration of pharmacological doses of DNP.

**Methods:**

We studied 14 healthy volunteers, divided into group A (n=4) and group B (n=10). At first, we studied four females (mean age: 57.3±4.5 y), three of whom underwent ^11^C-DNP PET scan at 2.5 h after the oral administration of 1 mg and 30 µg of DNP, respectively, while one patient was scanned following the oral administration of 30 µg of DNP (group A). Then, we studied five females and five males (48.3±6.1 y), who underwent ^11^C-DNP PET scan, without the oral administration of DNP (group B). Plasma DNP concentration upon scanning was measured by tandem mass spectrometry. Arterialized venous blood samples were collected periodically to measure plasma radioactivity and metabolites. In group A, ^11^C-DNP PET scan of the brain and whole body continued for 60 and 20 min, respectively. Subjects in group B underwent sequential whole-body scan for 60 min. The regional uptake of ^11^C-DNP was analyzed by measuring the standard uptake value (SUV) through setting regions of interest on major organs with reference CT.

**Results:**

In group A, plasma DNP concentration was significantly correlated with the orally administered dose of DNP. The mean plasma concentration was 2.00 nM (n=3) after 1 mg oral administration and 0.06 nM (n=4) after 30 µg oral administration. No significant difference in plasma radioactivity or fraction of metabolites was found between groups A and B. High ^11^C-DNP accumulation was found in the liver, stomach, pancreas, brain, salivary glands, bone marrow, and myocardium in groups A and B, in this order. No significant difference in SUV value was found among ^11^C-DNP PET studies after the oral administration of 1 mg of DNP, 30 µg of DNP, or no DNP.

**Conclusion:**

The present study demonstrated that the whole-body distribution of DNP after the oral administration of pharmacological doses could be evaluated by ^11^C-DNP PET studies, combined with the oral administration of DNP.

## Introduction

Donepezil hydrochloride (DNP) is an acetylcholinesterase inhibitor, prescribed for patients with Alzheimer’s disease (AD) (1). DNP is effective in preventing the progression of AD by blocking acetylcholine degradation in synapses of cholinergic transmission in the brain. DNP has adverse effects such as bradycardia, gastrointestinal symptoms, and acute pancreatitis. The adverse effects are related to the uptake of DNP in non-target organs with peripheral cholinergic innervations.

Although the pharmacokinetic study of DNP has been extensively studied in human, the whole-body distribution of this agent after oral administration remains unknown (2, 3). ^11^C-DNP positron emission tomography (PET) has been used to measure acetylcholinesterase density in the brain of normal subjects, patients with AD (4), and subjects with dementia and Parkinson’s disease (5). The ^11^C-DNP study was extended to assess whole-body cholinergic innervations in various organs of normal volunteers (6) and patients with Parkinson’s disease (7). These studies indicated that, ^11^C-DNP accumulates in the liver, pancreas, myocardium, bone marrow, colon, and salivary parotid glands after the intravenous injection of ^11^C-DNP tracer dose. However, the distribution of DNP after oral pharmacological administration remains unknown.

In case of DNP, it is difficult to study the whole-body distribution after the oral administration of ^11^C-DNP by means of PET scan, owing to the short physical half-life of ^11^C (20.33 min). The time to maximum peak plasma concentration (t_max_) of DNP after the oral administration was estimated at 5.2 h and 3.4 h in normal young (20-27 y) and elderly (65-82 y) subjects, respectively (3).

Given the slow absorption of ^11^C-DNP compared to its fast physical decay, radioactivity concentration after the oral administration of ^11^C-DNP would not be high enough for imaging the whole body (2, 3). In order to overcome this difficulty, normal volunteers in the present study were asked to take 1 mg and 30 µg of DNP orally. The ^11^C-DNP PET study was started at 2.5 h after the oral DNP intake by intravenous injection of ^11^C-DNP. During ^11^C-DNP PET data acquisition, ^11^C-DNP could trace the whole-body distribution of orally administered DNP.

Another concern in this study was the potential underestimation of ^11^C-DNP uptake due to the competitive uptake of DNP and ^11^C-DNP. A recent study estimated the dissociation constant (K_D_) of DNP to range from 17 to 39 nM in various pig tissues, measured through *in vitro* autoradiography (6). When the plasma concentration of orally administered DNP is close to or greater than K_D_, competitive uptake of DNP and ^11^C-DNP results in the reduction of ^11^C-DNP accumulation in the organs.

In human studies, the peak plasma concentration of DNP has been estimated at 8 nM at 4 hours after single oral administration of 2 mg of DNP (3). In order to evaluate the potential underestimation of ^11^C-DNP accumulation due to the high plasma DNP concentration of the oral pharmacological dose (1 mg), we performed additional ^11^C-DNP PET studies after the oral micro-dose (30 µg) administration of DNP. If the accumulation of ^11^C-DNP after the oral pharmacological dosing (1 mg) was equivalent to that reported after the oral micro-dose (30 µg) administration, underestimation of ^11^C-DNP accumulation due to the high concentration of DNP could not be taken into consideration.

We also measured the plasma DNP concentration during PET data acquisitions to compare it with the reported K_D_ values. In addition, we compared ^11^C-DNP PET scan without the oral administration of DNP with PET scans after the oral administration of DNP. Overall, the aim of the present study was to test the whole-body distribution of DNP after single oral dosing by means of ^11^C-DNP PET scan, following the intravenous injection of ^11^C-DNP tracer dose.

## Methods

### Subjects

A total of 14 normal volunteers were enrolled in the present study. The subjects were divided into group A, which was studied through ^11^C-DNP PET scan after the oral administration of DNP, and group B, which was evaluated by ^11^C-DNP PET scan after no oral administration of DNP. Group A consisted of four females (mean age: 57.3±4.5 y), three of whom underwent ^11^C-DNP PET scans twice. One PET scan was performed at 2.5 h after the oral intake of 1 mg of DNP and one at 2.5 h after the oral intake of 30 µg of DNP. In one subject, the study was performed only after 30 µg intake.

In the present study, we set the amount of DNP at 1 mg for the oral administration to reduce the probability of adverse effects in normal volunteers. This amount was 20% of the initially prescribed dose of DNP for therapy in clinical settings. No subject complained of the adverse effects after the oral administration of 1 mg of DNP.

The consensus guideline by the International Conference on Harmonization of Technical Requirements for Registration of Pharmaceuticals for Human Use (ICH) has approved less than 50% of the no-observed-adverse-effect level (NOAEL) in an exploratory clinical trial (11). Accordingly, DNP dose of 1 mg in the present study was within this range.

On the other hand, group B consisted of 10 normal volunteers (5 males and 5 females; mean age: 48.3±6.1 y). These subjects did not have any mental diseases and did not use any medicines regularly. The normality of their condition was checked by blood sampling, cranial magnetic resonance imaging, and electrocardiography. The whole-body dosimetry of ^11^C-DNP was reported to be 2.7 mSv for 500 MBq of ^11^C-DNP (6).

This study was approved by the Institutional Ethics Committee, and written informed consents were obtained from all the candidates.

### Preparation of ^11^C-DNP

We purchased donepezil hydrochloride from Tokyo Chemical Industry Co. Ltd. to be used as the standard for high-performance liquid chromatography (HPLC) in order to measure the specific activity of ^11^C-DNP products. Also, the desmethyl precursor of ^11^C-DNP, 2-((1-benzylpiperidin-4-yl)methyl)-5-hydroxyl-6-methoxy-2,3-dihydro-1H-inden-1-1-(5-O-desmethyl donepezil), was synthesized by NARD Institute Ltd. (Kobe, Japan).

Production of ^11^C-DNP was performed as previously described in the literature (8). In brief, the in-house cyclotron (CYPRIS HM18, Sumitomo Heavy Industry Co. Ltd., Tokyo, Japan) was employed to produce ^11^C by ^14^N(p, α)^11^C nuclear reaction with the irradiation of proton beam (25 µA and 18 MeV). ^11^C-carbon dioxide was converted to ^11^C-methyl triflate via ^11^C-methyl iodine.

Then, ^11^C-methyl triflate reacted with the precursor of ^11^C-DNP (5-O-desmethyl donepezil); ^11^C-DNP was purified by HPLC. The specific activity of ^11^C-DNP by the end of synthesis ranged from 84.7 to 510.3 GBq/μmol (mean: 151.8±95.2 GBq/μmol). The radiochemical purity was greater than 99.0%.

### Scan protocol and image reconstruction

### Group A

^11^C-DNP PET study in group A was performed, using the Eminence Sophia SET-3000 GCT/X scanner (Shimadzu Co., Kyoto, Japan) (9) under resting conditions with eyes closed. The PET study was conducted at 2.5-3.0 h after the oral dosing of DNP to coincide with the peak plasma level (t_max_= ~3.5 h) in healthy subjects (3).

The whole-body transmission scan for attenuation correction was performed for 7 min by means of ^137^Cs external point source. The sequential emission scan with three dimensional data acquisitions was started immediately after the intravenous injection of 270-370 MBq of ^11^C-DNP. The emission data were acquired over 60 min for the brain, followed by 20 min of whole-body scan, extending from the vertex to thighs.

The PET data were reconstructed, using the Dynamic Row-Action Maximum Likelihood Algorithm (DRAMA) (10). The standardized uptake value (SUV) images were obtained to correct the injected radioactivity and body weight; however, the whole-body CT scans were not acquired.

### Group B

The ^11^C-DNP PET scan in group B was performed, using the Eminence Sophia SET-3000 BCT/X scanner (Shimadzu Co., Kyoto, Japan) under resting conditions with eyes closed. The PET scan was initiated immediately after the injection of ^11^C-DNP solution, following a transmission scan with ^137^Cs as the external point source. All the images were reconstructed, using DRAMA (10) with an image matrix of 128×128, resulting in a voxel size of 4.0×4.0×3.25 mm^3^. The whole-body CT scans were acquired after emission scan for image fusion.

### Plasma radioactivity and metabolites

During the PET study, 12 arterialized venous blood samples (2 ml) were obtained at 0.5, 1.0, 1.5, 2.0, 2.5, 3.0, 5.0, 10.0, 20.0, 30.0, 45.0, and 60.0 min, respectively from a cubital vein and heated in a heating blanket. Arterialization of venous blood was confirmed by arterial oxygen saturation (> 89.4%) and arterial partial pressure of oxygen (> 52.5 mmHg) in 30.0 min samples in each subject.

The whole-blood radioactivity was measured in a well-type scintillation counter (Shimadzu Co, Kyoto, Japan). After measuring the whole-blood activity, the sample was separated into plasma and blood cell fractions by centrifugation (at 3000 g for 3 min at 4°C) to measure the plasma radioactivity. For metabolite analysis, a blood sample, taken at 30 min after injection, was centrifuged at 3000 g for 3 min at 4°C to obtain the plasma.

The plasma (2 ml) was denaturated with 1 M of HClO_4_:MeCN (7:3) and centrifuged at 3000 g for 3 min at 4°C. The supernatant solution was injected into a column (YMC ODS A-324, YMC Co., Ltd., Kyoto, Japan; 10 min i.d. × 30 cm long), with a solvent system of 0.1 M ammonium formate:acetonitrile (60:40) at a flow rate of 5.0 ml/min. The eluate was collected at a time interval of 0.5 min, and the radioactivity of the eluate in each collection vial was measured.

### Plasma concentration of DNP

After measuring the plasma radioactivity of blood samples, taken periodically during the scan, all the samples were collected to measure the plasma concentration of DNP. By means of HPLC (LC-20, Shimadzu Co., Ltd.) with a YMC-Triart C18 column (YMC Co.), the peak area for DNP was determined through tandem mass spectrometry (LC/MA/MS, API 5000, AB Sciex Pte. Co., Ltd.).

### PET data analysis

SUV images were obtained by normalizing the tissue concentration of ^11^C-DNP in terms of the injected dose and body mass. The region of interest (ROI) analysis was performed to evaluate the regional distribution of ^11^C-DNP. ROIs were placed on individual reconstructed axial, sagittal, and coronal PET images of the whole brain, myocardium, pancreas, and other organs in the CT scan (group B), using PMOD software version 3.4.0.4.

## Results

### Plasma DNP concentration

Table 1 summarizes the specific activity of ^11^C-DNP at injection, injected radioactivity, amount of intravenously injected ^11^C-DNP, and plasma concentration of DNP in each study. The plasma concentration of DNP was significantly correlated with the amount of orally administered DNP (P<0.01).

**Table 1:** Radioactivity, specific activity, and plasma concentration of DNP

Subject No.	Radioactivity (MBq)	Specific activity (GBq/µmol)	Oral DNP dose (µg)	Plasma DNP (nM)
Group A				
1-1	370	44	1000	2.550
2-1	370	90	1000	2.620
3-1	370	207	1000	2.524
1-2	332	57	30	0.068
2-2	270	108	30	0.102
3-2	370	65	30	0.097
4-2	295	46	30	0.067
Group B				
5	220	33	0	0.089
6	220	30	0	0.094
7	220	28	0	0.037
8	220	13	0	0.090
9	220	39	0	0.038
10	180	45	0	0.040
11	220	47	0	0.043
12	220	44	0	0.095
13	220	55	0	0.026
14	220	39	0	0.032

### Plasma ^11^C-DNP radioactivity

**Figure 1:**
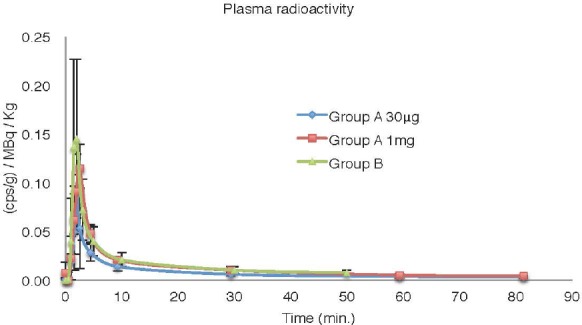
Plasma radioactivity during ^11^C-DNP PET for the Group A (1 mg and 30 mg oral administration studies) and the Group B (no oral DNP administration studies) corrected for injected radioactivity and body weight

Figure 1 illustrates the plasma radioactivity during ^11^C-DNP PET scan in group A (oral administration of 1 mg and 30 µg of DNP) and group B (no oral DNP administration), corrected for the injected radioactivity and body weight. The mean integrated radioactivity since injection (t=0) to 60 min was 0.64±0.27, 0.94±0.25, and 0.65±0.39 (cps/g)/MBq/kg for 1 mg dosing, 30 µg dosing, and no oral administration, respectively (Table 2).

**Table 2:** Plasma radioactivity and metabolite fraction

Subject No.	Integrated plasma radioactivity (cps/g)/MBq/Kg	Fraction of metabolite at 30 min (%)
Group A		
1-1	1.29	25.1
2-1	1.01	11.7
3-1	0.84	12.5
1-2	0.61	17.2
2-2	1.01	16.4
3-2	0.56	10.6
4-2	0.36	11.1
Group B		
5	0.95	13.4
6	0.85	30.7
7	0.24	12.0
8	0.36	-
9	0.37	28.9
10	0.89	23.7
11	0.75	-
12	1.51	35.5
13	0.41	29.7
14	0.19	34.5

No significant difference was found in the integrated plasma radioactivity among the three studies. The fraction of metabolite at 30 min following the ^11^C-DNP injection was not significantly different among the three studies (Table 2).

### Whole-body distribution of ^11^C-DNP

**Figure 2:**
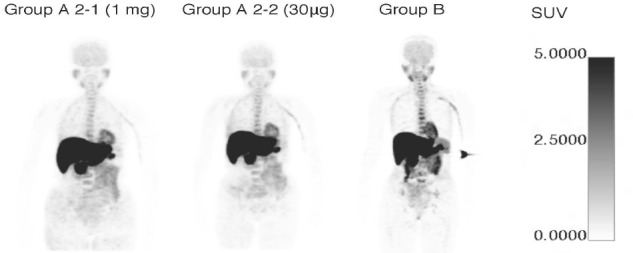
Whole body-SUV images for 1 mg, 30 µg, and no oral administration of DNP

Table 3 summarizes the mean SUVs at 60 min in various organs. There was no significant difference in SUVs of the evaluated organs between the study groups. Figure 2 illustrates the whole-body SUV images for 1 mg dosing, 30 µg dosing, and no oral administration of DNP.

**Table 3:** Mean SUV_mean_ for major organs in 1 mg-dosing, 30 µg dosing, and no dosing ^11^C-DNP PET study

Organs	mean SUV_mean_ after 1 mg-dosing	mean SUV_mean_ after 30 µg-dosing	mean SUV_mean_ after no dosing
Brain	0.9 ± 0.1	0.9 ± 0.1	1.4 ± 0.3
Salivary gland	1.9 ± 0.2	2.0 ± 0.3	2.5 ± 0.6
Lung	0.7 ± 0.1	0.7 ± 0.1	0.6 ± 0.3
Myocardium	2.9 ± 0.4	3.3 ± 0.7	3.9 ± 0.8
Liver	15.6 ± 0.8	12.9 ± 3.0	10.0 ± 1.7
Pancreas	6.5 ± 0.8	7.6 ± 0.9	8.7 ± 2.8
Bone marrow	2.3 ± 0.3	1.9 ± 0.1	1.9 ± 0.6
Muscle	0.5 ± 0.1	0.6 ± 0.1	0.4 ± 0.2
Colon	2.8 ± 0.7	3.4 ± 0.1	3.3 ± 1.3

Mean ± 1SD

## Discussion

The present study demonstrated that there was no significant difference in SUVs of various organs, plasma radioactivity of ^11^C-DNP (corrected for radioactivity decay), administered dose of DNP, body weight of the subjects, or metabolite fraction in the plasma among ^11^C-DNP PET studies (1 mg dosing, 30 µg dosing, and no oral administration of cold DNP) in normal volunteers.

Ohnishi et al. reported the plasma concentration of DNP after single oral administration of 2 mg of DNP in young and elderly subjects. The concentration linearly increased and reached the peak values of 3.4 and 3.1 ng/ml at 3.4 and 5.2 h, respectively (3). In the present study, the plasma concentration (1.2 ng/ml) at approximately 2.5 h after 1 mg oral dosing well corresponded to the reported values.

In this study, when 30 µg of DNP was administered orally, the plasma concentration was calculated to be 0.036 ng/ml. The results indicated that DNP concentration in circulating blood was proportional to the orally administered dosage. The present study demonstrated that ^11^C-DNP accumulation was not suppressed by single oral administration of 1 mg of DNP, as no difference in SUV was found between 1 mg and 30 µg of cold DNP orally administered.

Okamura et al. reported that ^11^C-DNP accumulation in the brain decreased after the daily oral administration of 5 mg of DNP for a period of six months (4). This was due to the increased plasma concentration of DNP after continuous dosing, as the mean DNP concentration in the plasma was reported to be 100 nM after daily oral administration (5 mg) for 18 days (11).

Although repeated 5 mg oral administration of DNP might suppress ^11^C-DNP accumulation due to the competitive uptake of orally administered DNP and intravenously administered ^11^C-DNP, single oral 1 mg dosing in the present study did not suppress ^11^C-DNP accumulation. Therefore, PET study after the intravenous injection of ^11^C-DNP could trace the whole-body distribution of DNP after single oral 1 mg dosing.

We recently reported the whole-body distribution of DNP in rats by means of ^11^C-DNP PET-CT scan (12). We found increased accumulation of ^11^C-DNP in adrenal glands, which was not reported in the present research or previous human studies. In our previous study, we also reported the slight accumulation of DNP in the myocardium and pancreas of rats, whereas the accumulation of DNP in these organs was elevated in humans in the present study. These findings indicate species differences in the whole-body distribution of DNP between humans and rats. In fact, species difference is recognized as one of the factors involved in the failure of new drug development.

The European Union and USA authorities have recommended the use of human PET studies, with labeled candidate compounds in the early phase of new drug development (13, 14). Although PET is expected to provide absorption, distribution, metabolism, and excretion (ADME) of candidate compounds in humans, pharmacokinetic study after oral dosing has not been yet reported. The present study demonstrated that whole-body ADME of DNP (after the oral administration of pharmacological dose) can be estimated by ^11^C-DNP PET study, combined with oral pharmacological dosing.

There were several limitations of the present study. Firstly, compartment analysis was not applicable due to changes in the specific activity of ^11^C-DNP in blood during the study. Overall, cold DNP and ^11^C-DNP, which are present in the circulating blood, show similar behaviors.

In a previous study, SUVs were proportional to the distribution volume, estimated by the compartment analysis (6). However, after the oral administration, the concentration of cold DNP increased within several hours; therefore, specific activity of ^11^C-DNP in circulating blood decreased. Compartment analysis is only applicable when the specific activity remains constant during the study (15). Therefore, we need to develop a quantitative method for conditions where the specific activity changes during the study.

Secondly, the cold DNP doses in oral administration were 1 mg and 30 µg, respectively. Also, the plasma concentrations were 2.00 and 0.06 nM, respectively. These values were below the K_D_ range (6-39 nM), reported in various organs in pigs (6). However, the plasma concentration over which the uptake of DNP from blood to tissue is suppressed has not been determined in humans.

Thirdly, the portion of unchanged ^11^C-DNP after injection was reported to be 82.5% at 30 min following the injection (4), 91% at 30 min after the injection (5), and more than 90% at 60 min after the injection (6). In the present study, it was 86.2% at 30 min after ^11^C-DNP injection in the 1 mg oral administration study, 83.5% in the 30 µg oral administration study, and 78.8% in the no oral administration study. These values were slightly lower than those reported values (4-6).

Two reasons have been noted for the observed discrepancy. First, our subjects were younger than the participants in the mentioned studies; therefore, metabolism was more active. Second, the administered radioactivity was smaller than previous reports; consequently, measurement errors might have occurred.

## Conclusion

In spite of the above-mentioned limitations, whole-body distribution of orally administered DNP was evaluated by PET scan after intravenous administration of ^11^C-DNP. The present design of ^11^C-DNP PET could be applicable to PET micro-dose studies for evaluating the whole-body pharmacokinetics of candidate compounds for the development of new drugs.
